# How mobile phone addiction leads to college students’ learning burnout: the role of depression as a mediator and fear of missing out as a moderator

**DOI:** 10.3389/fpsyt.2025.1569340

**Published:** 2025-06-02

**Authors:** Tianxiang Song, Hongze Zhu, Kaixu Yang, Wenhao Chang, Jianchao Ni

**Affiliations:** ^1^ Ningbo City College of Vocational Technology, International School, Ningbo, Zhejiang, China; ^2^ Xiamen University, School of Aerospace Engineering, Xiamen, Fujian, China; ^3^ College of Materials, Xiamen University, Xiamen, Fujian, China; ^4^ Ningbo University, School of Physical Science and Technology, Ningbo, Zhejiang, China; ^5^ Xiamen University, Institute of Education, Xiamen, Fujian, China

**Keywords:** mobile phone addiction, learning burnout, depression, fear of missing out, college students

## Abstract

**Background:**

With the widespread use of smartphones, mobile phone addiction is becoming increasingly common among college students, which has a negative impact on their learning. This study aims to explore how mobile phone addiction leads to college students’ learning burnout, with a focus on the mediating role of depression and the moderating role of FOMO (Fear of Missing Out).

**Methods:**

Convenient sampling was used to collect 1862 valid questionnaires from over 10 universities in China. A moderated mediation model was constructed to analyze the relationship and mechanism among mobile phone addiction, learning burnout, depression and FOMO through structural equation modeling.

**Results:**

(1) Mobile phone addiction has a significant positive impact on college students’ learning burnout (β=0.4767, p < 0.001); (2) Depression plays a partial mediating role between mobile phone addiction and learning burnout (95% CI= [0.0706,0.1145]), with the mediating effect accounting for 19.34% of the total effect; (3) FOMO moderates the relationship between depression and learning burnout. Specifically, depression has a stronger impact on learning burnout among college students with low FOMO.

**Conclusion:**

This study reveals the mechanism of mobile phone addiction on college students’ learning burnout, and confirms the mediating role of depression and the moderating role of FOMO. By integrating Self-Determination Theory, we further explain the specific mechanisms of FOMO’s moderating role. This offers a which provides a new perspective for understanding the impact of mobile phone addiction on college students’ learning burnout. It also provides a theoretical basis for colleges and universities to carry out mental health education and intervention.

## 1 Introduction

With the rapid development of mobile Internet technology, smartphones have evolved from a single communication tool to a multimodal medium that integrates social, entertainment and information acquisition functions. The popularity of smartphones has not only reshaped the mode of interpersonal communication, but also profoundly affected the learning habits and life rhythm of college students. As a landmark product of modern technology, smartphones have a significant trend of diversified functions, but the problem of mobile phone addiction caused by excessive use has become a major concern in the field of global public health (1). Mobile phone addiction is a behavioral addiction characterized by excessive smartphone use that leads to significant disturbances in daily life, including symptoms such as tolerance, withdrawal, and impaired impulse control (2). Studies have shown that about 25% of individuals have experienced or are currently dealing with mobile phone addiction, highlighting the urgency of mobile phone addiction as a public health issue (3). Among them, 34.9% of mobile phone users are aged between 18 and 30, indicating that college students have become a high-risk group for mobile phone addiction (J. X. 4).

In recent years, there is an increasing amount of research on the relationship between mobile phone addiction and mental health of college students. Studies have found that mobile phone addiction increases the risk of learning burnout through mediating mechanisms such as cognitive failure and difficulty in emotional regulation (5, 6). In addition, mobile phone addiction might also indirectly affect learning burnout through variables such as sleep quality and self-control ability (7). Specifically, mobile phone addiction not only takes up a lot of students’ time, leading to distraction and decreased sleep quality, but also weakens students’ self-control ability, thereby aggravating learning burnout (8). There are also studies exploring the relationship between mobile phone addiction and depression, examining the role of emotional and cognitive factors in the impact of mobile phone addiction on depression (9). In addition, Fear of Missing Out (FOMO), as a modern psychological phenomenon, has also gradually received attention in recent years. Some studies have explored the impact of FOMO on student learning burnout, examined the mediating role of mobile phone addiction and sleep quality, and explored the moderating role of mindfulness (10). At present, there is still relatively little research on how mobile phone addiction, depression and FOMO work together to cause learning burnout. Therefore, this study will further explore the relationship between these variables from the perspective of mediation and moderation. This analysis will not only help to deeply understand the impact mechanism of mobile phone addiction on learning burnout, but also provide a theoretical basis for colleges and universities to carry out mental health education and intervention.

### 1.1 Mobile phone addiction and learning burnout

Mobile phone addiction, also known as mobile phone dependence, refers to an individual’s excessive indulgence in mobile phone activities, which leads to a strong and continuous sense of dependence and craving for mobile phones, which in turn leads to impairment of social and psychological functions (11). In today ‘s highly developed Internet and communication technology, mobile phone addiction has become a common addictive behavior in society and has attracted much attention in the research of mental disorders, psychology and social behavior. Mobile phone addiction seriously threatens the academic performance and physical and mental health development of college students. Studies have shown that dependence on mobile phones is closely related to a variety of personal risk factors, among which learning problems, especially learning burnout, are the most prominent risk factors (P. S. 12; Joel 13). In recent years, empirical studies have shown that there is a significant positive correlation between mobile phone addiction and learning burnout. Mobile phone addiction takes up a lot of time, leading to distraction, poor sleep quality, and weakened self-control, thereby exacerbating learning burnout (9, 14).

The concept of learning burnout originated from the study of job burnout. In most study, the concepts of “learning burnout” and “academic burnout” are used interchangeably, as they both refer to a similar phenomenon characterized by emotional exhaustion, disengagement, and a lack of sense of achievement in academic contexts. However, there are subtle differences between the two. Learning burnout is more student-centered and captures their negative attitudes towards school courses and studies, including a decline in enthusiasm for courses and school activities, as well as a negative emotional state characterized by indifference and alienation towards classmates and friends (15). On the other hand, academic burnout is more focused on the broader educational environment and is often associated with academic stress, performance pressure, and institutional factors. In this article, we refer to learning burnout as a student-centered phenomenon that emphasizes the multifaceted nature of students’ experiences in the educational context. Learning burnout is mainly manifested in a three-dimensional structure of emotional exhaustion, cognitive disengagement, and lack of sense of achievement (16). For college students, learning burnout is manifested as a decrease in self-confidence in effectively managing academic obstacles, and then a general negative psychological tendency towards educational institutions, academic pursuits, and specific courses (17). Studies have shown that mobile phone addiction exacerbates learning burnout through multiple pathways. Its mechanism of action involves pathways such as cognitive resource depletion, emotional dysregulation, and behavioral adaptation disorders. Mobile phone addiction not only leads to cognitive failure and emotional exhaustion, but also indirectly affects college students’ learning burnout through a variety of psychological mechanisms (S. 18, 19). Mobile phone addiction might also affect learning burnout through the mediating effects of stress perception and self-control (20). In summary, mobile phone addiction consumes a lot of college students’ study, exercise and rest time, reduces their sleep quality, affects their learning motivation and learning efficiency, leads to poor academic performance, and then causes learning burnout. Based on this, the hypothesis is proposed:

H1: Mobile phone addiction has a positive impact on learning burnout.

### 1.2 The mediating effect of depression

Depression is a common mood disorder, with the core features of persistent negative emotional experiences and loss of interest. Studies have shown that individuals with depression might rely on mobile phones to relieve their negative emotions and spend more time on communication activities through mobile phones (21, 22), and excessive and problematic mobile phone use can lead to dependence (23). Mobile phone use might lead to various mental disorders, especially depression, anxiety, and sleep disorders (Sara 24). individuals with depression are at a higher risk of mobile phone addiction. Depressed individuals use smartphones more to relieve their negative emotions and also show phone checking behaviors to obtain social assurance, which increases the time they spend on smartphones (25). Studies have found that individuals with major depression have poorer coping strategies, higher levels of neuroticism, and stronger dependence on short-message (26), which are factors associated with mobile phone addiction. From the perspective of emotion regulation theory, excessive use of smartphones for emotional comfort can lead to social withdrawal and self-denial, thereby inducing or exacerbating depressive symptoms. Although this escape behavior can provide short-term emotional comfort, in the long run, it will aggravate emotional disorders and form a vicious cycle of “addiction-depression-burnout” (9). Related studies have investigated the correlation between psychological distress (such as depression) and learning burnout (27, 28). In addition, a meta-analysis showed that emotional problems, especially depression, are important predictors of poor academic performance in adolescents (29). Learning burnout is an important obstacle to students’ learning. It reduces the sense of meaning of learning and leads to psychological symptoms such as irritability, tension, inferiority and depression, which has a negative impact on their physical and mental health and personal development (W. 30). The compensatory Internet use theory points out that individuals addicted to mobile phones compensate for real frustrations through instant gratification, but long-term dependence exacerbates emotional disorders and becomes a catalyst for depression and burnout (14, 31). In summary, depression is not only one of the potential consequences of mobile phone addiction, but also an important predictor of learning burnout. Based on this, the hypothesis is proposed:

H2: Depression mediates the relationship between mobile phone addiction and learning burnout.

### 1.3 The moderating effect of FOMO

As a modern psychological phenomenon, FOMO has gradually attracted attention in recent years. FOMO is a personality trait whose core feature is the desire to maintain continuous social interaction and the fear of missing important social experiences (32). FOMO is closely related to a sense of social disconnection and internalizing problems such as depression and anxiety symptoms (33–35). According to Self-Determination Theory (36), individuals with high FOMO experience heightened frustration of their basic psychological needs (autonomy, competence, and relatedness) due to excessive social comparison and compulsive connectivity (37) This intensifies their susceptibility to depression, making them more vulnerable to learning burnout when facing stressors. Social Comparison Theory (38) further explains that FOMO-driven individuals engage in upward comparisons, exacerbating feelings of inadequacy and emotional dysregulation, thereby amplifying depression’s impact on burnout.​​ High levels of FOMO can cause students to feel worried and uneasy when facing learning tasks, thereby distracting their attention and reducing their learning efficiency. Specifically, students with high levels of FOMO are more susceptible to the negative effects of mobile phone addiction, which increases the likelihood of cognitive failure. For example, students might check their phones frequently for fear of missing important learning resources or social activities, thereby reducing their attention to learning content (C. 39).

At the same time, FOMO can also exacerbate learning burnout through indirect pathways. Students with high levels of FOMO are more susceptible to the negative effects of mobile phone addiction, which increases the risk of cognitive failure and emotional exhaustion, and ultimately leads to learning burnout (20). According to the I-PACE model (Integrative Procedure for Assessing the Components of Experience), FOMO as an external stimulus may amplify the effect of depression on learning burnout by intensifying negative emotional responses (40; W. 41). Thus, FOMO not only heightens the emotional responses associated with depression but also creates a feedback loop where learning burnout further increases feelings of FOMO, leading to a cycle of distress. Negative coping styles are positively correlated with anxiety levels (42). The cognitive behavioral model also points out that individuals with high FOMO are overly sensitive to social cues and tend to maintain social connections through continuous mobile phone use, thereby amplifying the impact of depression on academic burnout (1). On the contrary, low FOMO individuals are less constrained by virtual social interactions, and their depression is more likely to be directly transformed into academic alienation. Therefore, FOMO might play a moderating role between depression and learning burnout. Based on this, the hypothesis is proposed:

H3: FOMO moderates the relationship between depression and learning burnout among college students.

**Figure 1:**
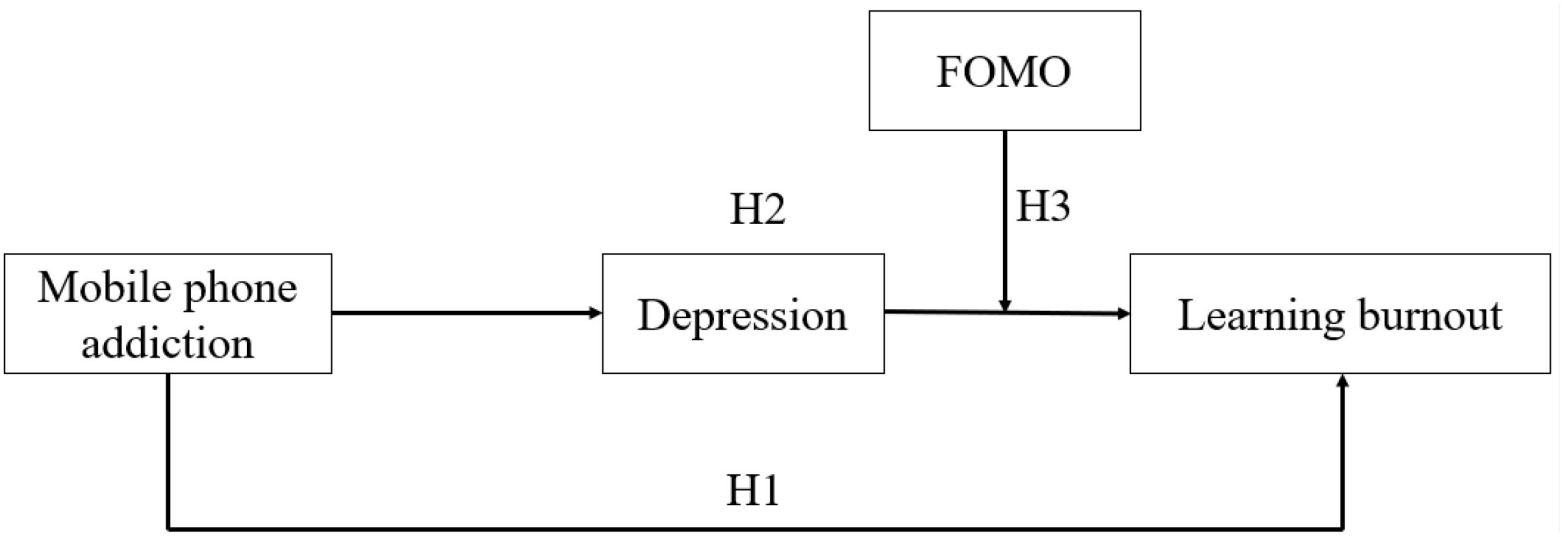
Hypothesized model of moderated mediation. H1: Mobile phone addiction → Learning burnout. H2: Mobile phone addiction → Depression → Learning burnout. H3: Depression → FOMO → Learning burnout.

This study proposed a moderated mediation model (**Figure 1**) to explore the relationship and mechanism between mobile phone addiction, learning burnout, depression, and FOMO. The main purpose is twofold: to explore among college students (1) the mediating role of depression between mobile phone addiction and learning burnout, and (2) the moderating role of FOMO between depression and learning burnout.

## 2 Methods

### 2.1 Data sources and sample characteristics

This study used a convenient sampling method and covered over 10 colleges and universities of different levels and regions in China. A questionnaire survey was conducted among college students to gather data. In the questionnaire design stage, questions that might directly link to personal identity, such as names, addresses or other personal identifiers, were strictly avoided. Before formally collecting data, it was clearly stated to students that the study would be conducted anonymously and voluntarily to ensure the privacy of the participants, and the informed consent of the participants was obtained. A total of 2000 questionnaires were distributed, and 138 invalid questionnaires (e.g., those with regular answers or incomplete responses) were excluded. The effective collection rate of the questionnaire was 93.1%. The basic characteristics of the sample are as follows: 764 males (41.0%), 1098 females (59.0%); 425 urban residents (22.8%) and 1437 rural residents (77.2%). The age range of the participants was 17 to 23. The sample is relatively evenly distributed in terms of demographic variables, with a certain representativeness, and can better meet the needs of this study.

### 2.2 Research tools

This study used four scales to measure the key variables. When evaluating the reliability, validity and model fit of the scale, the following statistical parameters were mainly used: Cronbach’s α coefficient was used to evaluate the internal consistency of the scale; KMO (Kaiser-Meyer-Olkin) test was used to evaluate the applicability of factor analysis. RMSEA (root mean square error of approximation) was used to measure the model error, and its value below 0.08 indicated that the model fit was good. CFI (comparative fit index), GFI (goodness of fit index), and NFI (standardized fit index) were used to evaluate the overall fit of the model, and its value above 0.8 indicated that the fit was good. The χ²/df (chi-square/degree of freedom) ratio should usually be less than 3, for large sample sizes, this indicator can be used as a reference and needs to be combined with RMSEA, CFI, GFI, NFI and other indicators for comprehensive judgment. It should be noted that these thresholds are not fixed, and their applicability might vary depending on the research background and sample size, which should be reasonably interpreted according to specific circumstances.

#### 2.2.1 Mobile phone addiction scale

The mobile phone addiction scale compiled by L. Chen was used (43). This scale has a high degree of recognition and is widely used in the field of mobile phone addiction research. The scale has 12 items. The Likert 5-point scoring method was used for scoring, with 1 point representing “strongly disagree” and 5 points representing “strongly agree”. The higher the score, the higher the degree of agreement with the question. The KMO value of the scale was 0.932, and the research data is suitable for extracting information; the Cronbach’s α coefficient of the scale was 0.926, and the scale had good consistency, and the measurement results were valid. The items were summed up and the mean was taken to obtain the variable of mobile phone addiction. The higher the score, the higher the degree of mobile phone addiction.

#### 2.2.2 Learning burnout scale

The Chinese version of the Learning Burnout Scale was used (44), which comprised of 20 items covering three domains of burnout: dejection (8 items), improper behavior (6 items), and reduced personal accomplishment (6 items). Items were selected according to the actual situation of college students. The scale in this research has 11 items. The Likert 5-point scoring method was used for scoring, with 1 point representing “completely inconsistent” and 5 points representing “very consistent”. The higher the score, the higher the degree of agreement with the question. The KMO value of the scale was 0.945, and the research data was suitable for extracting information; the Cronbach’s α coefficient of the scale was 0.919, and the scale had good consistency, and the measurement results were valid. The items were summed up and the mean was taken to obtain the variable of learning burnout. The higher the score, the higher the degree of learning burnout.

#### 2.2.3 FOMO scale

FOMO scale compiled by LI Qi was used (45). This scale is adapted from the original FOMO scale developed by Przybylski (32), and has been validated for use among college students. Items were selected according to the actual situation of college students in China. The scale has 4 items, with typical items such as “I fear that others might have more exciting experiences or achievements than me”. The Likert 5-level scoring method was used for scoring, with 1 point indicating “completely inconsistent” and 5 points indicating “very consistent”. The higher the score, the higher the degree of agreement with the question. The KMO value of the scale was 0.813, and the research data was suitable for extracting information; the Cronbach’s α coefficient of the scale was 0.927, and the scale had good consistency, and the measurement results were valid. The items were summed up and the mean was taken to obtain the variable of FOMO. The higher the score, the higher the degree of FOMO.

#### 2.2.4 Depression scale

The depression subscale from the Depression Anxiety Stress Scales (DASS-21) was used (46). It has 7 items. The Likert 4-point scoring method was used for scoring, with 1 point representing “completely inconsistent” and 4 points representing “very consistent”. The higher the score, the higher the degree of agreement with the question. The KMO value of the scale was 0.910, and the research data was suitable for extracting information; the Cronbach’s α coefficient of the scale was 0.919, and the scale had good consistency, and the measurement results were valid. The items were summed up and the mean was taken to obtain the variable of depression. The higher the score, the higher the degree of depression.

## 3 Data processing

SPSS26, AMOS26 and PROCESS macros were used for statistical analysis. Firstly, confirmatory factor analysis was conducted on the scale to test the reliability and validity level of the scale. Subsequently, descriptive statistics and correlation analysis were performed on the data. Finally, Model 4 and Model 14 of the PROCESS macro program written by Hayes were used (47), Model 4 was selected to test simple mediation, while Model 14 was used to examine moderated mediation, aligning with our hypotheses, and the bias-corrected percentile Bootstrap method was used to test the significance of the mediating effect. If the 95% confidence interval did not contain a value of 0, the effect was significant and had statistical significance (48). To avoid bias in moderation effects, all variables were standardized beforehand.

Common methodological bias might arise when data is collected using self-reporting methods. Before formal data analysis, the Harman single-factor method was used to test for common method bias (49). The results (**Table 1**) showed that there were 4 factors with characteristic roots greater than 1, and the total variance explained by the first common factor was 38.67%, which was less than the critical value of 40%. Therefore, the data of this study did not have common method bias.

**Table 1:** Initial eigenvalues.

Element	Total	Percentage of Variance	Accumulation %
1	13.15	38.67	38.67
2	3.56	10.48	49.15
3	2.79	8.20	57.35
4	1.72	5.06	62.41

## 4 Research results

### 4.1 Confirmatory analysis and correlation analysis

To ensure the reliability and validity of the research tool, a confirmatory factor analysis containing four first-order factors was first conducted. The results showed that the fit index of the measurement model was good: χ2/df = 9.465, p < 0.001, RMSEA = 0.067, CFI = 0.898, GFI = 0.843, NFI = 0.888. As shown in **Table 2**, the loading values of each factor item are all greater than 0.50 (p < 0.001). The AVE values of each factor are all greater than 0.50, and the square root of AVE (the underlined numbers on the diagonal line) exceeds the correlation coefficient between factors, indicating that the discriminant validity of the scale is good. The Cronbach’s α coefficient and combined reliability of each factor are all greater than 0.70, indicating that the reliability of the factor is good. Overall, the reliability and validity of this research tool are satisfactory, and the survey data are suitable for further analysis.

**Table 2:** Descriptive statistics and correlation coefficient matrix of each variable (N = 1862).

Variable	1	2	3	4	Cronbach ‘s α	CR	AVE	CFA Loading Range (mean)
Mobile phone addiction	0.718				0.926	0.927	0.516	(0.565-0.841,0.714)
Learning burnout	0.568 ^**^	0.714			0.919	0.919	0.510	(0.619-0.787,0.712)
FOMO	0.460 ^**^	0.429 ^**^	0.872		0.927	0.927	0.761	(0.803-0.930,0.871)
Depression	0.461 ^**^	0.437 ^**^	0.563 ^**^	0.806	0.919	0.928	0.649	(0.714-0.854,0.804)
Mean	3.303	2.923	2.648	1.712				
SD	0.920	0.772	1.013	0.677				

* indicates p<0.05, indicates p<0.01, and * indicates p<0.001.

The scale average is the sum of the scores of each question in the variable divided by the number of questions; CR is the combined reliability, AVE is the average extracted variance, the triangular matrix below the table is the Pearson correlation coefficient between the variables, and the diagonal is the square root of the average extracted variance (AVE).

Correlation analysis was conducted on the main variables of mobile phone addiction, learning burnout, depression, and FOMO. Considering that the variables are continuous variables, the Pearson correlation coefficient test was used. The results (**Table 2**) show that there is a significant positive correlation between mobile phone addiction, learning burnout, depression, and FOMO. Specifically, mobile phone addiction is positively correlated with college students’ learning burnout (r = 0.568, p<0.01); mobile phone addiction is positively correlated with FOMO (r = 0.460, p<0.01); mobile phone addiction is positively correlated with depression (r = 0.461, p<0.01); college students’ learning burnout is positively correlated with FOMO (r = 0.429, p<0.01); college students’ learning burnout is positively correlated with depression (r = 0.437, p<0.01); college students’ learning burnout is positively correlated with depression (r= 0.563, p < 0.01). The results of correlation analysis lay a foundation for the subsequent test. In order to eliminate the impact of collinearity on the research results, collinearity diagnosis was performed on the predictor variables before testing for mediation and moderation effects. The diagnostic results show that the variance inflation factors of all predictor variables (1.372, 1.582, and 1.585 respectively) are lower than the standard threshold of 5. The data of this study does not exhibit serious collinearity problems and is suitable for further testing.

### 4.2 Test of mediation effect

Firstly, a mediation model was constructed with learning burnout as the dependent variable, depression as the mediating variable, and mobile phone addiction as the independent variable. Referring to Wen Zhonglin’s mediation effect test procedure (50), Model4 (Model4 is a simple mediation model) in the SPSS plug-in macro PROCESS compiled by Hayes (47) was used for regression analysis (the number of Bootstrap sampling was set to 5000) to test the mediating effect of depression between mobile phone addiction and learning burnout among college students. The specific results are shown in the table below (**Table 3**).

**Table 3:** Mediating effect of depression.

Variable	Learning burnout		Depression		Learning burnout	
	β	t	β	t	β	t
Mobile phone addiction	0.4767***	29.6368	0.3491***	23.348	0.3845***	21.6768
Depression					0.2640***	10.9064
R-sq	0.3231		0.2386		0.3639	
F	221.6015***		145.4839***		212.3313***	

* indicates p<0.05, indicates p<0.01, * indicates p<0.001; β is the standardized coefficient; R-sq is the adjusted R-square.

Mobile phone addiction has a significant positive predictive effect on college students’ learning burnout (β= 0.4767, t=29.6368, p<0.001), and hypothesis H1 is supported. After adding the mediating variable depression, mobile phone addiction has a significant positive prediction effect on depression (β= 0.3491, t=23.348, p<0.001), and depression has a significant positive prediction effect on college students’ learning burnout (β= 0.2640, t =10.9064, p<0.001), mobile phone addiction still has a significant positive prediction effect on college students’ learning burnout (β = 0.3845, t=21.6768, p<0.001). With reference to Wen Zhonglin’s mediation effect test, it can be considered that depression plays a mediating role between mobile phone addiction and college students’ learning burnout, and hypothesis H2 is supported.

Secondly, to further verify the mediating effect of depression, the bootstrap sampling method (sampling times 5,000 times) was used to obtain the bootstrap test results of the mediation effect, which are organized into the following table (**Table 4**). The results show that the confidence interval of the indirect effect includes the value 0, and the confidence interval of the direct effect includes the value 0, that is, the indirect effect exists, and the direct effect exists, indicating that depression plays a partial mediating role. The proportion of the mediation effect is 19.34%.

**Table 4:** Decomposition of mediation effect.

Effect	Effect Size	Boot Standard Error	LLCI	ULCI	Relative Effect Size
Total Effect	0.4767	0.0161	0.4451	0.5082	
Direct Effect	0.3845	0.0177	0.3497	0.4193	80.66%
Indirect effects	0.0922	0.0113	0.0706	0.1145	19.34%

### 4.3 Moderating effect test

Third, FOMO was included as the moderator variable into the mediation model, and a moderated mediation model was constructed. Model 14 in the SPSS plug-in macro PROCESS was used to conduct a moderated mediation effect test, and the following table was obtained (**Table 5**). After adding the moderator variable FOMO, mobile phone addiction positively predicts depression (β = 0.3395, t = 22.4321, p < 0.001), depression positively predicts learning burnout (β = 0.4397, t = 6.4267, p < 0.001), and mobile phone addiction positively predicts learning burnout (β = 0.4397, t = 6.4267, p < 0.001). Mobile phone addiction still significantly predicts learning burnout (β=0.3601, t=-20.0392, p<0.001). There is a positive and significant relationship between FOMO and learning burnout (β=0.2298, t=6.6951, p<0.001). The interaction term between depression and FOMO is significant in the model (β=-0.0783, t=-4.0932, p<0.001), that is, the interaction term can significantly affect learning burnout. Therefore, FOMO moderates the relationship between depression and learning burnout, and hypothesis H3 is supported.

**Table 5:** Test of moderated mediation effect.

Variable	Depression		Learning Burnout	
	β	t	β	t
Mobile phone addiction	0.3395***	22.4321	0.3601***	20.0392
Depression			0.4397***	6.4267
FOMO			0.2298***	6.6951
Depression * FOMO			-0.0783***	-4.0932
R-sq	0.2129		0.3799	
F	503.1974***		284.4662***	

* indicates p<0.05, indicates p<0.01, * indicates p<0.001; β is the standardized coefficient; R-sq is the adjusted R-square.

Finally, in order to interpret the moderating effect of FOMO between depression and learning burnout more vividly, the mean of the moderating variable FOMO plus or minus one standard deviation was used as the grouping standard to obtain the moderating effect decomposition table (**Table 6**). At the same time, the effects of depression on learning burnout under high FOMO and low FOMO conditions were depicted and a simple slope test was performed.

**Table 6:** Decomposition table of the moderating effect of FOMO.

FOMO	Effect Size	Boot Standard Error	BootLLCI	BootULCI
eff2-eff1	-0.0275	0.0091	-0.0461	-0.0107
eff3-eff1	-0.0551	0.0181	-0.0923	-0.0214
eff3-eff2	-0.0275	0.0091	-0.0461	-0.0107

BootLLCI refers to the lower limit of the 95% interval of Bootstrap sampling, BootULCI refers to the upper limit of the 95% interval of Bootstrap sampling.

**Figure 2:**
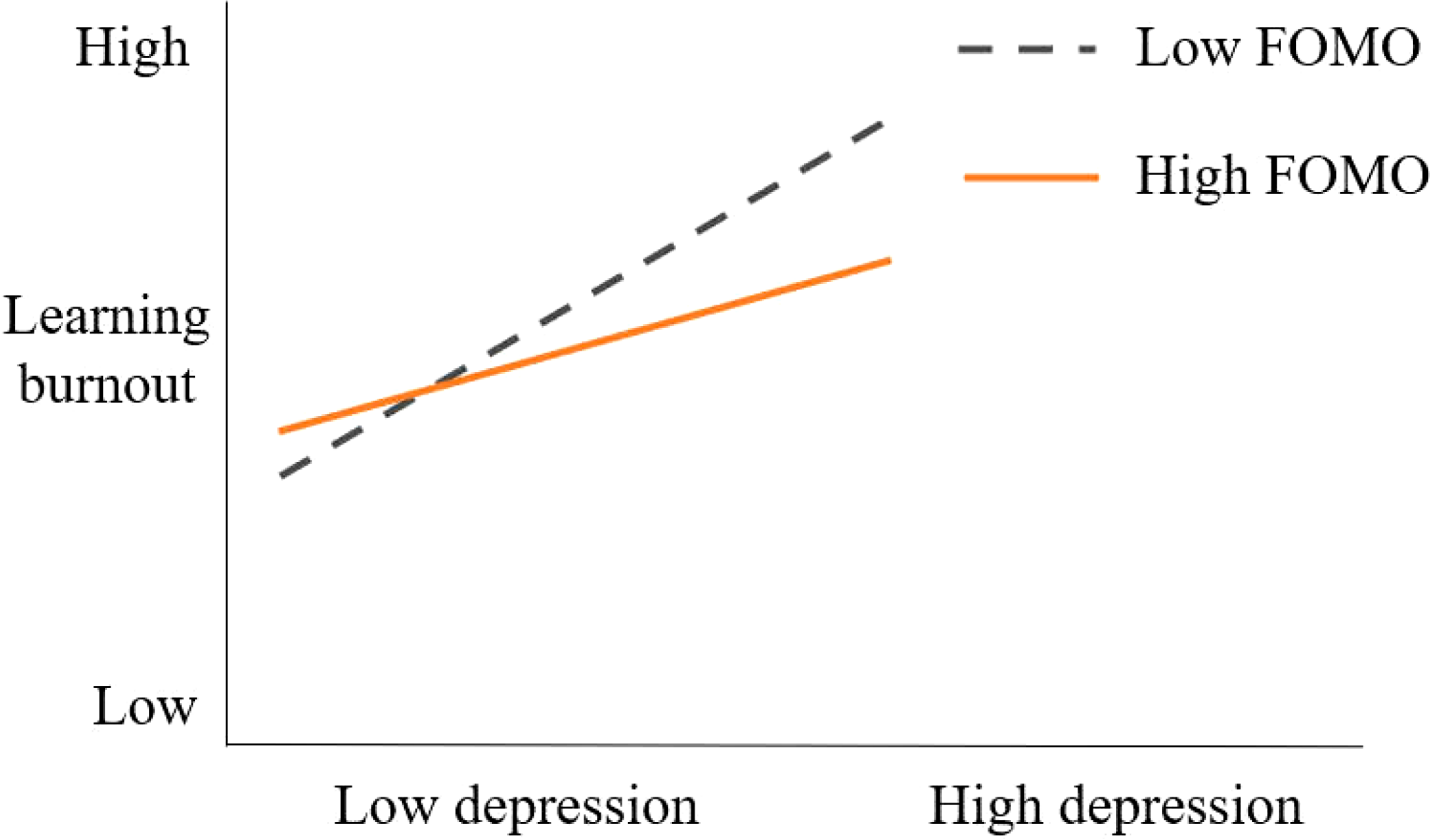
Simple slope graph.

**Figure 2** shows that under different levels of moderating variables, depression has a positive effect on college students’ learning burnout (the slopes are all greater than 0). Overall, as the level of depression increases, the learning burnout of college students with low FOMO increases faster, indicating that depression has a stronger impact on learning burnout among college students with low FOMO. Hypothesis H3 is further supported.

## 5 Discussion

### 5.1 The impact of mobile phone addiction on learning burnout

This study reveals that mobile phone addiction has a significant positive impact on college students’ learning burnout. In the digital age, smartphones have been deeply integrated into college students’ daily lives, and the addiction caused by their excessive use has affected students’ learning process. From the perspective of direct impact, mobile phone addiction causes students to be frequently distracted when studying and to find it difficult to focus on academic tasks. A large amount of time is spent on non-learning activities such as browsing mobile phones, playing games, and browsing social media, which directly reduces the effective time and energy used for learning, leading to low learning efficiency and delayed learning progress. This is one of the direct causes of learning burnout. In terms of indirect impact, mobile phone addiction is often accompanied by the intensification of academic procrastination behavior (51). Addicted students are prone to rely on the instant gratification brought by mobile phones. When faced with learning tasks, they tend to postpone the start time or interrupt the learning process to use mobile phones. In the long run, students will have a strong sense of fatigue and powerlessness when facing heavy academic work, which further promotes the breeding of learning burnout (52). In view of this, when dealing with the problem of learning burnout among college students, educators should pay attention to the factor of mobile phone addiction, comprehensively consider its direct and indirect impact paths, and deeply explore the potential psychological mechanisms, so as to develop more accurate and effective intervention strategies to guide students to use mobile phones reasonably and reshape healthy learning habits.

### 5.2 The mediating role of depression

The study found that depression partially mediates between mobile phone addiction and learning burnout, with the mediating effect accounting for 19.34% of the total effect (95% CI = [0.0706, 0.1145]). This conclusion reveals the internal psychological mechanism of mobile phone addiction affecting learning burnout, that is, mobile phone addiction not only directly affects learning burnout, but also indirectly affects learning burnout by inducing depression. In the context of mobile phone addiction, students are addicted to the virtual world, gradually disconnect from real learning life, reduce social interactions, lose learning motivation, and then experience learning burnout. Students who are in a state of learning burnout for a long time are likely to fall into the quagmire of depression under the continuous influence of multiple external factors such as academic pressure, social difficulties and self-expectation gap. Once depression occurs, it will react to mobile phone addiction behavior, forming a vicious circle (53). Students in a depressed state might be more inclined to seek comfort and escape reality with the help of mobile phones, further aggravating the degree of addiction and making the learning burnout worse. Understanding this mediating mechanism is of great significance for breaking the vicious cycle, reducing the risk of mobile phone addiction, and formulating scientific prevention and treatment strategies. Educators should carry out targeted psychological counseling and intervention activities based on this, such as cultivating students’ positive coping styles and emotional regulation abilities, helping them to rebuild their interest and confidence in learning, guiding them to use mobile phones moderately, gradually relieving depression, reducing learning burnout, and achieving a benign transformation of their learning status.

### 5.3 The moderating effect of FOMO

This study also found that FOMO plays a moderating role in the relationship between depression and learning burnout. Grounded in Self-Determination Theory, which posits that individuals are motivated by the need to satisfy their basic psychological needs for competence, autonomy, and relatedness (36), we can understand how FOMO influences students’ responses to depression. Specifically, when facing depression, college students with low FOMO lack the incentive or constraint mechanism brought by the worry about missing opportunities, which diminishes their motivation to engage in learning activities. This aligns with SDT, as their basic psychological needs may not be sufficiently met, making it difficult to effectively resist the negative impact of depression on their learning status. Therefore, when the degree of depression increases, the degree of learning burnout will rise faster. College students with high FOMO are more worried about missing learning opportunities and poor grades. According to Social Comparison Theory, individuals with high FOMO are likely to engage in upward social comparisons, which can heighten their anxiety but also serve as a motivational force (38). This anxiety becomes a motivation to a certain extent, prompting them to work hard to maintain their learning status when they are depressed, and the speed of learning burnout is relatively slow. The moderating effect of FOMO shows that students with different psychological states have different responses to learning burnout when facing depression. This finding provides a new perspective for the understanding and intervention of college students’ learning behavior. In educational practice, differential guidance strategies can be implemented for students with different levels of FOMO.

### 5.4 Research implications

This study highlights the significant impact of mobile phone addiction on learning burnout among college students, with depression acting as a mediator and FOMO as a moderator. While the findings are based on a sample from a specific region in China, it is important to consider the cultural context of the study. In Chinese culture, which is characterized by collectivist values, the fear of missing out (FOMO) may be particularly pronounced. Collectivist cultures often emphasize social harmony, group cohesion, and the importance of maintaining social connections. This cultural context may amplify the impact of FOMO on learning burnout, as students may feel a stronger need to stay connected and avoid missing out on social interactions.

Considering these findings, it is imperative for school counselors and educational personnel to be vigilant in identifying students who may be at risk. Possible indicators include signs of excessive mobile phone use, symptoms of depression, and expressions of fear of missing out. Educational institutions could consider implementing policies that limit mobile phone usage during class times and encourage more interactive and engaging teaching methods to reduce reliance on digital devices. Furthermore, there is a need for collaboration between educational institutions and mental health providers to develop targeted interventions aimed at addressing mobile phone addiction, enhancing emotional regulation skills, and promoting healthier coping mechanisms. Such collaborative efforts are essential to mitigate the adverse effects of mobile phone addiction on students’ academic performance and overall well-being. Additionally, universities and colleges can play a proactive role by conducting educational outreach programs to inform students about the negative effects of excessive mobile phone use on mental health and academic performance. These programs can include workshops, seminars hat highlight the importance of balanced technology use and provide strategies for managing mobile phone addiction. By integrating these measures into the educational environment, institutions can create a supportive atmosphere that encourages students to develop healthier habits and reduce the risk of learning burnout.

## 6 Limitations and prospects

This study has certain limitations. Firstly, the study adopted a cross-sectional data design and convenient sampling method. Although the sample is diverse to a certain extent, it might not fully represent the entire college student population due to sampling bias, and the causal relationship between variables cannot be determined. Secondly, the sample was drawn from colleges and universities in China, and there might be certain geographical and cultural limitations. The majority of participants were from urban and rural areas within a specific region of China, and the sample may not fully represent the diverse cultural and socioeconomic backgrounds of college students across the country, and the results may not be directly applicable to students from other regions or cultural backgrounds. In particular, the cultural context of collectivism in China may influence the manifestation and impact of FOMO. In addition, the study mainly relies on self-report data, which might have subjective biases, such as social desirability bias. While the Harman single-factor test indicated that common method bias was not a significant issue in this study, it is still important to acknowledge that self-report measures can introduce potential social desirability bias and common method variance. At the same time, the study only considered two factors, depression and FOMO, while other potential variables (such as self-efficacy, social support, etc.) might also affect the relationship between mobile phone addiction and learning burnout.

Future research can be carried out in the following directions: Firstly, adopt a longitudinal research design to track college students’ mobile phone usage behavior, psychological state and learning situation at different time points to more accurately reveal the causal relationship between variables. Secondly, expand the sample range to cover college students from different countries and cultural backgrounds, and adopt a more rigorous sampling method to enhance the universality and representativeness of the research results. In addition, combine multiple evaluation methods, such as behavioral observation, interviews, longitudinal designs and experimental design, to reduce the bias caused by a single data source and obtain more comprehensive and accurate research results. Additionally, further explore other possible mediating and moderating variables, such as self-efficacy, social support, etc., to build a more complete theoretical model. At the same time, future research should also explore the differences between educational use of mobile phones (such as accessing educational content) and general use (such as scrolling through social media), and their respective roles in learning burnout.

## 7 Conclusion

By constructing a moderated mediation model, this study reveals the relationship between mobile phone addiction, depression, FOMO and learning burnout in college students and their mechanisms of interaction. It is found that mobile phone addiction not only directly leads to learning burnout among college students, but also indirectly affects learning burnout through the mediation of depression. In addition, FOMO plays a moderating role between depression and learning burnout. These findings provide a more comprehensive perspective for understanding the impact of mobile phone addiction on college students’ academic performance, and also provide a theoretical basis for colleges and universities to carry out mental health education and intervention.

## Data Availability

The raw data supporting the conclusions of this article will be made available by the authors, without undue reservation.
